# The clinical application of microincision vein harvesting of the great saphenous vein in coronary artery bypass grafting

**DOI:** 10.1186/s12872-020-01555-5

**Published:** 2020-06-17

**Authors:** Shao-Zhong Zhang, Guo-Xiang Wang, Xiao-Tong Zhou

**Affiliations:** Department of thoracic-cardiovascular, Affiliated Hospital of Xuzhou Medical University, No. 58 of Hubei Street, Yongan District, Xuzhou, 221000 China

**Keywords:** Microincision, Bridge vessel of great saphenous vein, CABG

## Abstract

**Background:**

The present study aimed to summarize the clinical application of microincision vein harvesting (MVH) of the great saphenous vein in coronary artery bypass grafting (CABG).

**Methods:**

From July 2014 to October 2017, 160 patients underwent coronary artery bypass grafting. Among them, 80 patients received MVH of the great saphenous vein, and 80 received open venous harvesting (OVH). The results of the sampling operation, complications during hospitalization, and the long-term patency of the great saphenous vein were compared between the two groups.

**Results:**

All the patients in both groups received successful operations. The difference in the length of the veins obtained and the injury of the veins was not statistically significant (*P* > 0.05). The difference in the long-term patency rate of the graft vessels between the two groups was not statistically significant. The in-hospital mortality rate was the same in both groups. The MVH group had noticeable advantages over the OVH group in terms of the vein collection times, the incision length, and the complications experienced when performing the leg incisions (*P* < 0.01). The time relating to the patients’ observed early out-of-bed activity was significantly longer in the MVH group. Furthermore, the patients’ hospitalization length was significantly shorter in the MVH group compared to the OVH group (*P* < 0.05). The MVH group had significant advantages in pain score and patient satisfaction, and this difference was also statistically significant (*P* < 0.05).

**Conclusions:**

The MVH procedure met the requirements of CABG in vein grafting. When compared with OVH, MVH can significantly reduce leg incision complications and improve patients’ overall satisfaction with their hospital experience.

## Background

Coronary heart disease (CHD) is a serious risk to an individual’s health and wellbeing with a continuously increasing incidence rate. Treatments of CHD mainly comprise drug treatment, stent implantation, and surgical treatment. Furthermore, CHD patients undergo coronary artery bypass grafting (CABG) when they experience medical treatment failures and the recurrent challenge of unstable angina. CABG is the most effective surgical procedure for the treatment of CHD. This is particularly evident for patients who are experiencing ineffective results from their medical treatment for severe coronary artery trunk disease, severe stenosis of triple-vessel, or severe diffuse stenosis of double-vessel [1, 2]. In particular, these patients undergo a CABG procedure when their coronary angiography reveals more than 50% stenosis of the left main trunk or more than 75% stenosis of two vessels, more than 75% stenosis of three vessels or multiple diffuse lesions [3].

The vessels used for bypass grafting in CABG are mainly the internal thoracic artery, great saphenous vein (GSV), radial, or gastroepiploic artery. In China, it is more common to use the GSV [4]. However, the incidence of graft vessel obstruction, poor wound healing, and sensory skin abnormalities caused by open vein harvesting (OVH) of the GSV is relatively high [5]. At present, some hospitals in major cities are gradually promoting the method of harvesting the GSV under endoscopy, which presents important advantages and disadvantages. The main disadvantages include high wastage and high operation costs, which make it difficult for the procedure to be carried out in many prefecture-level municipal tertiary hospitals. Microincision vein harvesting (MVH) is a recently emerging surgical technique, where the main operation is to release GSV from two ends of the microincision. MVH of the GSV has the advantages of being a treatment that only involves a small trauma region, so ensures a fast recovery, a short duration procedure, well-performed incisions, and a low operation cost [6]. MVH of the GSV is considered similar to no-touch technology [7, 8]; therefore, it is gradually being adopted in many hospitals in China. MVH is additionally becoming the preferred method as a result of the simple equipment used in MVH technology, mainly including head-mounted lights, venous hooks, and titanium metal clips. Another advantage is that an endoscope is not used during this procedure. Emerging evidence shows that MVH of the GSV has more clinical benefits than methods of traditional incisions for harvesting the GSV, as this method is safer and has significantly lower complications of the wound [9–11].

To evaluate the beneficial characteristics of MVH over OVH, we used both surgical techniques on CABG patients. The prognostic indexes, including reducing venous vascular injury, leg complications, and adverse psychological conditions in patients after the operation, were studied retrospectively.

## Methods

### Patients

From July 2014 to October 2017, 160 patients who underwent CABG were recruited. The study protocol was approved by the Medicine Ethics Committee and was conducted in accordance with the Declaration of Helsinki. Furthermore, all patients provided informed consent prior to enrollment into the study.

### Study design

These patients were divided into two groups: the MVH group and the OVH group (*n* = 80, both). Patients in the MVH group were treated with MVH, and patients in the OVH group were treated with OVH. Patients requiring emergency surgery, or who had severe varicose veins of the lower extremities, or who required total artery bypass were excluded (the clinical data is presented in Table 1).

**Table 1:** Clinical data of the MVF and the OVF groups (x ± SD, n)

Clinical index	MVF group(*n* = 80)	OVF group(*n* = 80)
Age (years)	63 ± 9	61 ± 7
Gender		
Male	60	46
Female	20	34
BMI (kg/m^2^)	25.4 ± 3.8	24.6 ± 4.5
Left ventricular ejection	12	14.0
fraction(< 0.50)		
Three-vessel lesions of coronary artery	68	60
Diabetes	43	38
Varicose veins of the lower extremities	4	5

Note: *MVF* microincision vein freeing, *OVF* open vein freeing

All the comparisons between the two groups were *P* > 0.05

### Interventions

**Fig. 1:**
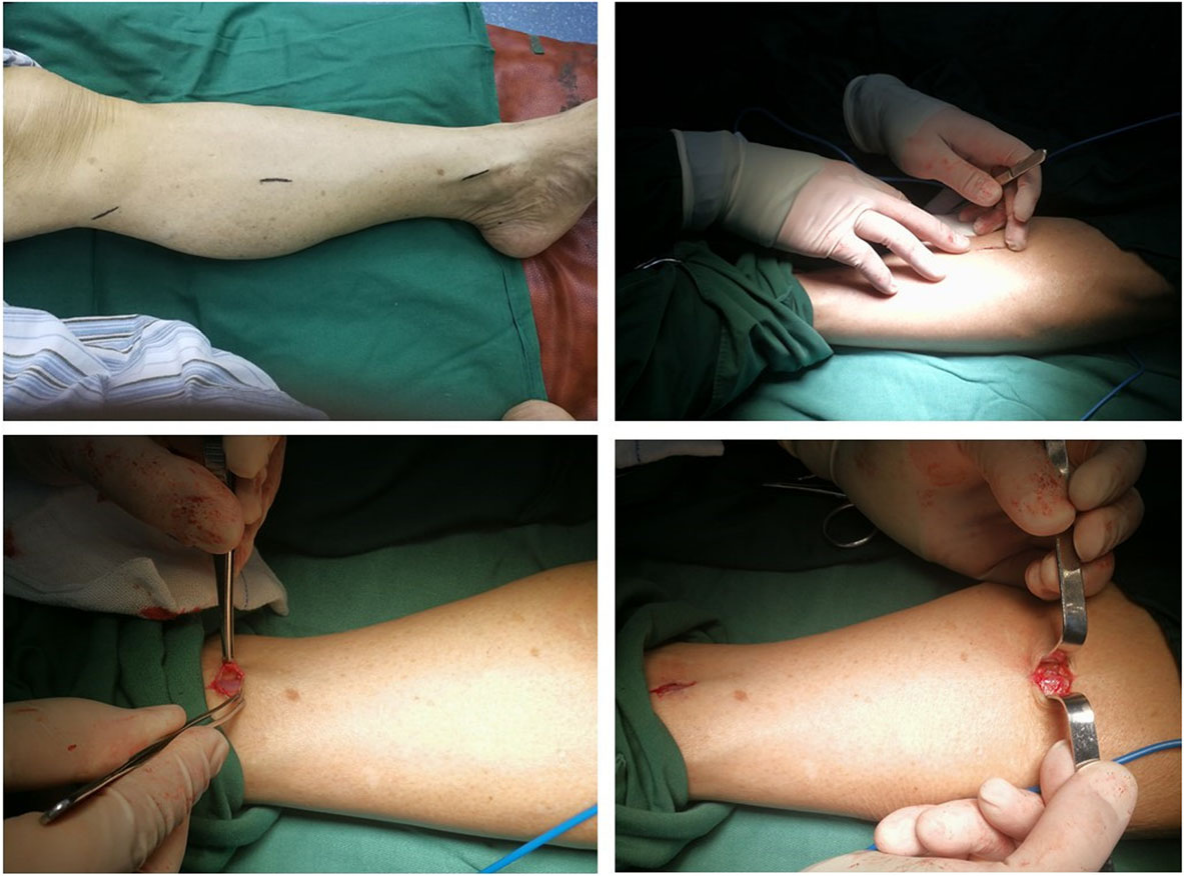
Intraoperative photograph of the microincision vein harvesting technique

In the MVH group, head-mounted surgical magnifiers, titanium metal clips, and two venous hooks were used for the operation. The MVH procedure includes mostly fine operations, so surgeons consider it a learning curve [12]. During the operation, incorrect actions can damage the intimal epithelium of venous vessels, induce autologous coagulation dysfunction, or result in poor autologous remodeling, causing the possibility of early stenosis or occlusion of venous bypass vessels [13]. Therefore, in selecting the GSV, we need to follow the principle that the ratio of the diameter of the bypass vessel freed to the diameter of the target vessel is ≤2.8; bypass vessels are smooth and have few collateral branches [7, 13, 14]. Meanwhile, skilled surgical techniques and a clear understanding of vascular anatomy are prerequisites for obtaining complete, smooth, and high-quality veins. There were three commonly performed microincisions in the MVH group, including the incision above the ankle joint (1.5 cm), the incision below the knee joint (1.5 cm), and the incision between the knee and the ankle joint (2 cm) (Fig. 1). The GSV from above the ankle joint to below the knee joint was efficiently freed. Two venous hooks were implanted into the incision above the ankle joint, and the anterior and posterior space of the GSV was freed. Furthermore, in the region near the distal end of the collateral branch, the branches were amputated by electrocoagulation with a long electric knife, and then the GSV was gradually freed. Thereafter, the vein trunk was amputated and ligated below the knee joint, and the GSV was pulled out. The proximal collateral end of the vein was clamped with titanium clips. Under direct inspection, the veins were expanded, and the branches were processed for future use.

Conversely, in the OVH group, conventional surgical instruments and routine surgical techniques were used to complete the operation. In short, this included performing an incision on all skin in the affected region along with completing the projection of the GSV on the surface. Thereafter, the vein was freed, and the branches were ligated. The vein was additionally retained for future use.

GSVs freed by the two methods were used as bypass vascular bridges to anastomose target vessels with conventional CABG techniques. In simple terms, the patient lay in a supine position, with single-lumen endotracheal intubation and vein anesthesia applied and the chest and lower extremities exposed. Thoracotomy was performed in the middle of the sternum, and the free internal mammary artery was used as an anterior descending branch bridge vessel to reveal the diseased coronary vessels. The free bridge vessel with the distal end of the anterior descending branch, blunt edge branch, and posterior descending branch, the proximal end of the bridge vessel, and the ascending aorta line-by-side were anastomosed. After exhausting the blood vessels in the bridge and checking for no bleeding, the patency of the bridge blood vessels can be detected with an arterial blood flow meter.

### Data collection

During the operation, ultrasound was used to locate bypass vessels for flow monitoring to ensure the patency of venous graft vessels. The time, length, and injury of the veins obtained were also recorded during the procedure. After the operation, the incision and complications, including incision exudation, skin ecchymosis, subcutaneous hematoma, incision infection, and lower limb edema, were observed and recorded. The preoperative cardiac function, the degree of coronary artery disease, the proportions of diabetes mellitus and varicose veins of the lower extremities, the time of early ambulation, hospitalization length, and the in-hospital mortality rate were compared between the two groups. During the in-hospital and postoperative follow-ups, the visual analog scale was used to evaluate the degree of pain experienced by the patients.

A retrospective study of the surgical techniques between the two groups was performed within 4 years after CABG, based on the long-term patency rate of the GSV and the results of the follow-up session. The presence and degree of stenosis of the graft vessels were analyzed by coronary computed tomography angiography (CTA) or coronary angiography, and stenosis of a 50% vascular diameter was used as the standard: > 50% stenosis indicated poor long-term patency.

### Statistical analysis

The data was statistically analyzed using the statistical software SPSS19.0. Count data was expressed as the frequency (rate/proportion) and evaluated using an X^2^-test. The measurement data was expressed as mean ± standard deviation (x ± SD) and evaluated using the t-test. In addition, normally distributed data was expressed as median and evaluated using a rank-sum test. A result of *P* < 0.05 was considered as statistically significant.

## Results

### Demographic data

There were no significant differences in age, gender, or body mass index between the MVH group and the OVH group (*n* = 80, both) (Table 1). All the patients in both groups received successful operations.

### Perioperative outcomes

The time for harvesting the vein and the length of the incision was shorter in the MVH group. Complications in patients’ legs after the operation were also fewer in the MVH group than in the OVH group, and these differences were statistically significant (*P* < 0.01). The difference in the rupture of veins needing suture between the two groups was not statistically significant, whereas the length of veins obtained in both groups was similar (*P* > 0.05). There was no significant difference in the length of veins freed and the number of wounded sites in the veins between the two groups; therefore, the differences were not statistically significant (*P* > 0.05). The rates of incision infection, lower limb edema, and hospitalization stay were greater in the OVH group than in the MVH group (*P* < 0.05, Table 2).

**Table 2:** Comparison of surgical results between the MVF group and the OVF group (x ± SD, n)

Clinical index	MVF group(*n* = 80)	OVF group(*n* = 80)	P
Operative time (min)	21.5 ± 4.0	36.8 ± 6.5	< 0.01
The length of veins freed (cm)	32.8 ± 5.8	34.2 ± 7.2	> 0.05
The number of wounded sites in the veins	1.1 ± 0.6	1.2 ± 0.7	> 0.05
Total length of skin incision	3.6 ± 0.3	24.2 ± 5.6	< 0.01
Complications of leg incision			
Incision exudation	0	8	< 0.01
Skin ecchymosis	7	9	> 0.05
Skin numbness	9	32	< 0.05
Incision infection	0	4	< 0.05
Lower limb edema	5	21	< 0.05
Time to get out of bed after operation(h)	42.0 ± 4.1	27.0 ± 5.6	< 0.05
Hospitalization stay (day)	7.2 ± 1.3	10.4土2.1	< 0.05
Leg wound pain score(0–10)	2.0 ± 1.1	3.6 ± 2.2	< 0.05

### Angiographic outcomes

**Fig. 2:**
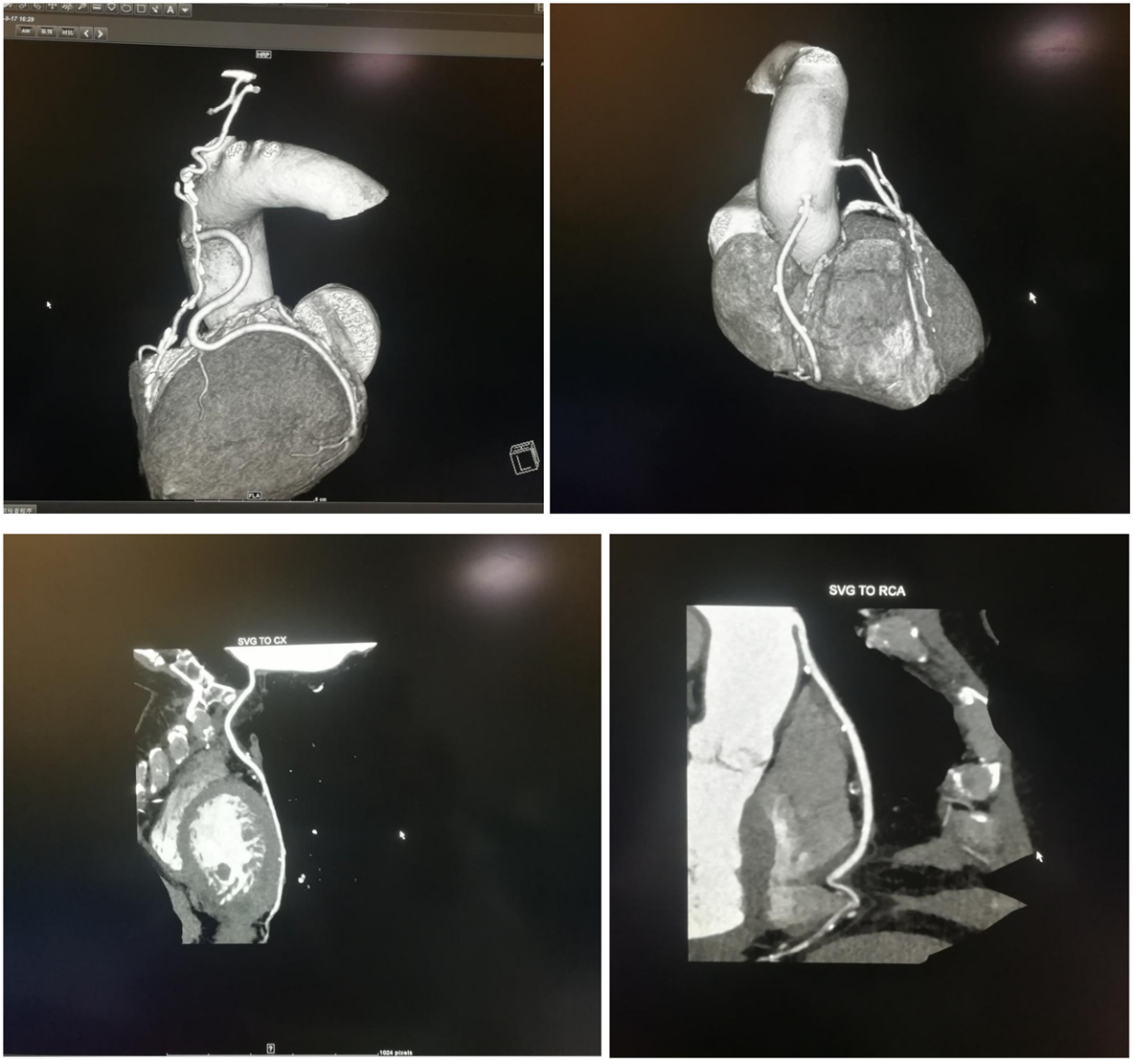
Vascular CTA after CABG in MVH

**Fig. 3:**
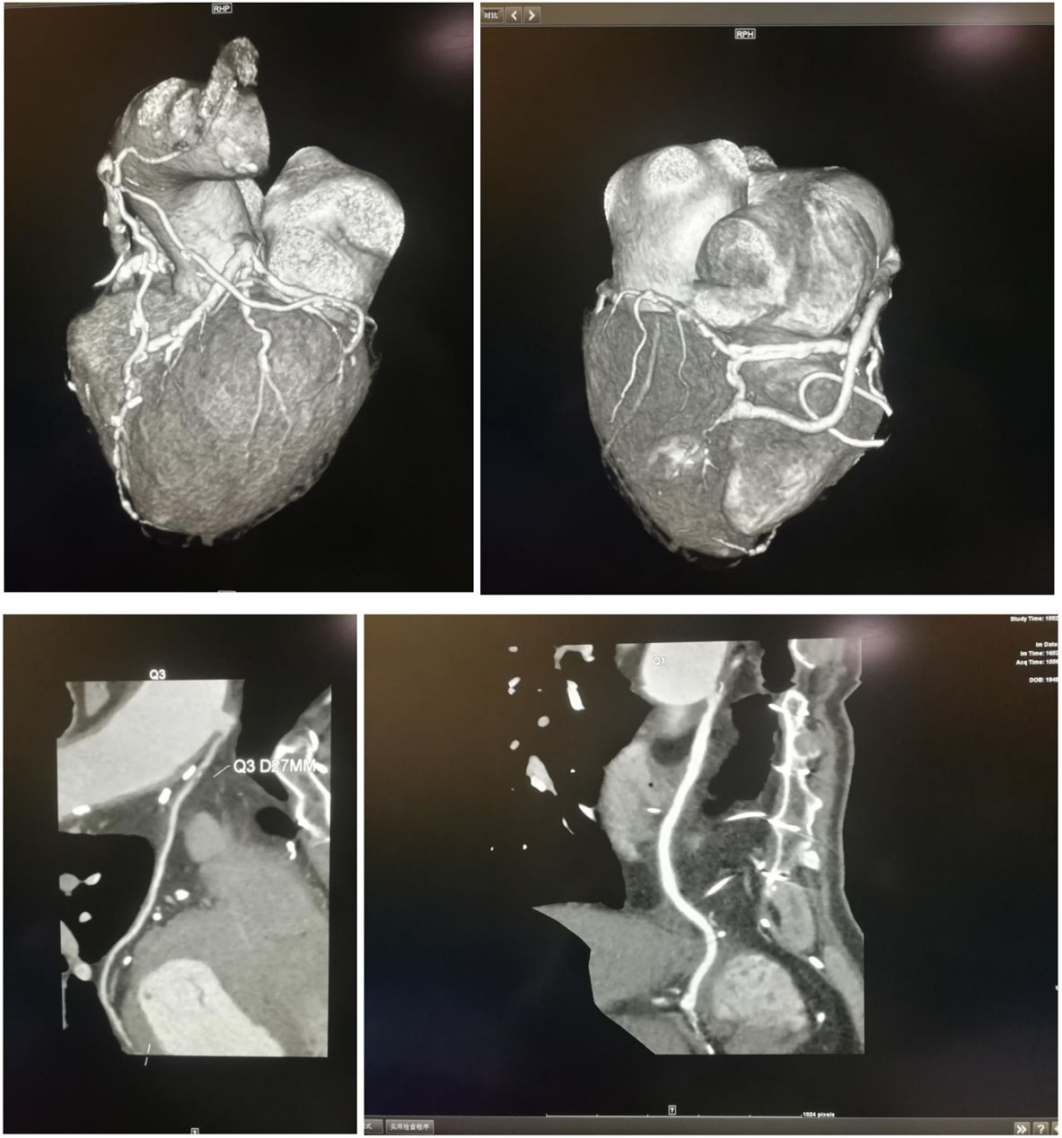
Vascular CTA after CABG in OVH

Two methods were evaluated by angiography. The results are as follows (Fig. 2 and Fig. 3)**.**

### Follow-up results

The long-term patency rate of the GSV was compared, resulting in a lack of significant statistical difference observed. The difference in the long-term patency of graft vessels between the MVH group and OVH group was not statistically significant; the difference in the degree of vascular injury between the two groups was also not statistically significant (Table 3).

**Table 3:** Comparison of long-term patency of bridging vessels between the MVF group and the OVF group within four years of follow-up sessions

Saphenous vein sampling method	Patency of great saphenous vein		χ^2^	P
	Pipe diameter > 50%	Pipe diameter < 50%		
MVF	75	5	0.754	0.385
OVF	72	8		

## Discussion

In this study, we compared the results of the sampling operation, complications during hospitalization, and the long-term patency of the great saphenous vein between the two groups. Results showed that the MVH group had noticeable advantages over the OVH group in terms of the vein collection times, the incision length, and the complications experienced when performing the leg incisions (*P* < 0.01). Furthermore, the patients’ early out-of-bed activity, hospitalization length, pain score, and patient satisfaction in the MVH group were significantly better than in the OVH group (*P* < 0.05).

The MVH group had significant advantages over the OVH group in the time taken to obtain the veins, the incision length, and the vein quality (*P* < 0.01). These differences were statistically significant. The OVH method using an endoscope mainly collects veins from the free saphenous vein above the knee to the groin. It is difficult to place the endoscope under the knee, due to the excessive subcutaneous tension of the calf. It may also damage the saphenous vein intima, which is not conducive to obtaining veins compared with the MVH method. Moreover, the cost of endoscopic vein collection is high, the steps are cumbersome, and the available value MVH group has the same effect. In the MVH group, the freed blood vessels met the requirements of conventional CABG by presenting a shortened operation time, accelerated early out-of-bed activity of patients after the operation, and the reduced psychological burden affecting patients. For obese patients, lower extremity subcutaneous tissue is relatively loose, providing an easily established surgical tunnel. However, in patients with thin and tight skin, many small branches of vessels present the challenge of smaller surgical tunnels resulting in the inability of carbon dioxide to fill the subcutaneous space to induce subcutaneous emphysema. This increases the difficulty of the operation to a certain extent, thus affecting the quality of the veins. In the MVH group, surgeons should operate carefully and have a clear understanding of vascular anatomy. Titanium metal clips or electrocoagulation hooks should be used to close, ligate, and cut off the distal end of the adventitia and branches of venous vessels. Surgeons should attempt to avoid damaging the adventitia or angulation and prevent the forming of manual stenosis, which may cause a low long-term patency rate.

A follow-up study was carried out based on long-term follow-ups of 160 patients with CABG, using CTA or coronary angiography. The patency of graft vessels in the two GSV groups collected by the two methods within 4 years after the operation was analyzed and studied. The results revealed that the difference in the degree of stenosis of graft vessels between the MVH group and the OVH group was not statistically significant and the degree of injury to vascular intima in the two groups was similar. These further revealed that the vascular injury or long-term patency of freed GSV between the two groups were alike.

The incidences of leg wound pain, exudation, edema, incision infection, and cutaneous nerve injury were significantly lower in the MVH group than in the OVH group (*P* < 0.05). These results revealed that after MVH, patients subjectively reported feeling a small incision, rapid recovery, no bleeding, no obvious numbness, and normal activity when compared with conventional OVH. This, in turn, improved their satisfaction with their hospital treatment. The results of the present study further confirm the feasibility and authenticity of previous studies. MVH has a significant effect and is worthy of popularization. Furthermore, in the MVH group, fewer traumas and less bleeding were observed in the subcutaneous incision. Three incisions were less than 2 cm, and the incidence of lower-limb edema was also much lower than in the OVH group. This suggests that MVH can also reduce the compression bandage time. In the OVH group, varying degrees of complications occurred in leg wounds in approximately one-third of the patients, which affected early activities and prolonged their hospitalization stay [15].

There are some limitations to MVH. First, although there is no significant difference between the two in terms of vascular patency on the bridge angiography after CABG, further study is required into the molecular changes of venous intima damage, and whether there is any effect on shortening the use of clinical antiplatelet aggregation drugs. Second, for some elderly patients, due to the excessive number of branches in their great saphenous veins, deep and large venous sinuses, excessively thin blood vessel diameters, and other objective factors, MVH acquisition may need to be converted to conventional methods. Finally, MVH is more difficult to master and involves something of a learning curve. It may take some time to obtain the saphenous vein from three small microincisions under certain conditions.

## Conclusions

This study revealed that the MVH operation used simple surgical equipment at a cost-effective price. Furthermore, after a significant period of knowledge acquisition and practice of the MVH procedure, high levels of procedural success can be achieved, while reducing the disadvantages of a complicated procedure, the high cost, and the large equipment wastage to a significant extent when compared to harvesting the vein under an endoscope [12, 16]. Patients are also more willing to accept less painful, less expensive, and less invasive surgical methods.

## Data Availability

The datasets generated and/or analysed during the current study are not publicly available due to the lack of an online platform but are available from the corresponding author on reasonable request.

## Abbreviations

CABG: Coronary artery bypass grafting
CHD: Coronary heart disease
GSV: Great saphenous vein
OVF: Open vein freeing
MVF: Microincision vein freeing
CTA: Computed tomography angiography
SD: Standard deviation
